# Impact of scale of aggregation on associations of cardiovascular hospitalization and socio-economic disadvantage

**DOI:** 10.1371/journal.pone.0188161

**Published:** 2017-11-28

**Authors:** Ivan C. Hanigan, Thomas Cochrane, Rachel Davey

**Affiliations:** 1 Spatial Epidemiology Group, Centre for Research and Action in Public Health, Health Research Institute, University of Canberra, Canberra, ACT, Australia; 2 University Centre for Rural Health, Sydney Medical School, University of Sydney, Sydney, NSW, Australia; University of Missouri Columbia, UNITED STATES

## Abstract

**Background:**

There are numerous studies that show an increased incidence of cardiovascular disease with increasing levels of socio-economic disadvantage. Exposures that might influence the relationship include elements of the built environment and social systems that shape lifestyle risk behaviors. In Canberra (the Australian capital city) there has been a particular housing policy to create ‘mixed-tenure’ neighborhoods so that small pockets of disadvantage are surrounded by more affluent residences (known as a ‘salt-and-pepper’ pattern). This may contribute to a scatter of higher incidence rates in very small areas in this population that may be obscured if aggregated data are used. This study explored the effect of changing the scale of the spatial units used in small area disease modelling, aiming to understand the impact of this issue and the implications for local public health surveillance.

**Methods:**

The residence location of hospitalized individuals were aggregated to two differently scaled area units. First, the Australian Bureau of Statistics Statistical Area 2 (SA2) which is normally used as the basis for deidentification and release of health data. Second, these data were aggregated to a smaller level: the Statistical Area 1 (SA1). Generalized Additive Models with penalized regression splines were used to assess the association of age-sex-standardized rates for cardiovascular disease hospital admissions with disadvantage.

**Results:**

The relationships observed were different between the two types of spatial units. The SA1 level exposure-response curve for rates against the disadvantage index extended in a linear fashion above the midrange level, while that found at SA2-level suggested a curvilinear form with no evidence that rates increased with higher disadvantage beyond the midrange.

**Conclusion:**

Our result supports findings of other work that has found disadvantage increases risk of cardiovascular disease. The shape of the curves suggest a difference in associations of cardiovascular disease rates with disadvantage scores between SA1 versus SA2. From these results it can be concluded that scale of analysis does influence the understanding of geographical patterns of socio-economic disadvantage and cardiovascular disease morbidity. Health surveillance and interventions in Canberra should take into account the impact of the scale of aggregation on the association between disadvantage and cardiovascular disease observed.

## Introduction

Cardiovascular disease incidence may vary geographically, due to the spatial variation of the determinants of these diseases. Understanding the pattern requires spatial data analysis. But a well-known problem exists in analysis of any health data that analyses at an inappropriate scale may obscure or distort the relationships investigated.

The problem of finding different results at different scales is well-recognised. There may be different statistical results at different levels, and sometimes severe bias may attenuate toward the null or even the opposite relationship may be found in analyses across different scales [1, 2]. Openshaw [3] coined the term ‘Modifiable Areal Unit Problem’ (MAUP) to define it. The observed relationships of any spatial data analysis may differ substantially between analyses of the same data, aggregated to different areal units. This is due to differences between the aggregated information content (e.g. levels of signal and noise).

The MAUP consists of two sub-problems; the scale of aggregation issue and the zoning configuration issue [4]. The scale issue is a problem when the aggregation units used are at an inappropriate resolution (granularity) for the phenomenon under investigation. The scale issue can arise when analyses are performed at levels of aggregation that may not be at appropriate scale to capture the true variation in the outcome of interest, and the exposures to risk factors contributing to this [5]. The zonation issue is a problem when areal units aggregate data into different patterns than the underlying phenomena due to their artefactual shapes. In some instances there is a mismatch created between the outcome data and the spatial pattern of the exposures which may obfuscate the association found in statistical models. It has been suggested that the use of smaller spatial units is likely to mitigate these issues because these allow flexible aggregation in a spatial hierarchy [6]. This can result in data that are more sensitive to the underlying spatial pattern of the outcome response and the driving exposures. In experimentation with simulated point patterns Kang et al. [7] showed that different results were observed and different inferences would be made when analysing data at different spatial scales. In this study we analysed empirical data based on areal units, to extend our knowledge of the impact of this issue. Our study question specifically relates to the common practice of aggregating point data into areal units prior to release for researchers, because releasing point data is considered a privacy and ethical risk. We use observed data to demonstrate the effect this has on the ability to detect and describe the association between cardiovascular disease and disadvantage.

This issue is very important for health surveillance systems managers around the world. Currently the effect of the MAUP is inherent in the spatial units used in health data dissemination in many countries, including in Australia, due to the misguided aggregation of locational identifiers in an attempt to maintain privacy and confidentiality of individuals. This practice may easily confound any associations observed, and lead to incorrect cross-level inference, and poor public health outcomes. This is known as the so-called ‘ecological fallacy’, a concept that has been used to describe the inappropriate inference of individual-level (and small-area) associations from aggregate-level results [8]. This can introduce errors, bias and contradictions to the understanding of exposures causing disease.

The important processes determining the successful analysis of geographical health data therefore involve both the interaction between individuals, society and the living and non-living environments on the one hand, and the protection of individual identity, confidentiality and security on the other. In this paper, we focus primarily on the scale issue since this is a critical first step in analysis. The issues of zonation, and how best to represent the residual variation within the spatial structures used is the subject of ongoing research [5].

In Canberra the impact of the scale issue may be large for any observed associations between cardiovascular disease and socio-economic disadvantage because this city is well-known for its pockets of socioeconomically disadvantaged residents, surrounded by more affluent residents [9, 10]. Cardiovascular disease in Canberra neighbourhoods have also been investigated at high spatial resolution and findings suggest that disease incidence varies at small area levels, possibly related to factors such as walkability [11, 12].

### The ecology of cardiovascular disease

Cardiovascular disease (CVD) is an important cause of morbidity and mortality in Australia. The Australian profile derived from the Global Burden of Disease (GBD) 2015 ranks some categories of CVD as causing the most premature deaths [13]. Risk factors such as smoking are well known [14]. However, other less understood risk factors include social disadvantage [15], physical activity patterns [16, 17], and atmospheric pollution [18]. The mechanisms by which disadvantage influences CVD morbidity can be contextualized using the interconnected elements of population composition, behavior and habitat [19]. Population composition includes the age, sex and genetic characteristics of the people. Behaviors, such as diet and physical activity influence risk factors. Habitat includes the configuration of the urban environment that encourages (or limits) access to amenities that lead to physical activity. One of the salient features of cardiovascular disease progression is atherosclerosis. Genetic and metabolic processes influence this universal process creating some disparity between sub-population groups. The behavior element incorporates the way humans interact with one another and with their environment. These include smoking, physical activity, and diet. Cultural differences between different groups of people will drive different behavior patterns and thus the exposure and susceptibility of individuals to these cardiovascular health risks. In the habitat element there are many environmental factors implicated with cardiovascular disease including: urban design, transport, atmospheric pollution; meteorological systems; water quality; and aspects of landscape geochemistry [19].

### Socio-economic disadvantage

A variety of epidemiology literature focuses on health inequalities caused by the differences in poverty, deprivation, and disadvantage [20, 21]. There is often an inverse association evident in morbidity and mortality rates with Socio-Economic Status (SES). This may be due to a variety of individual level risk factors such as diet or smoking, which are influenced by SES. Other explanations refer to the contextual attributes of disadvantaged neighbourhoods. This understanding is further complicated by findings that it is not just simple material wealth or deprivation, but relative status and hierarchy that are involved [22]. In Australia, statistical analyses have found differences between SES groups in the rates of various causes of death, including CVD [15] as well as with life-expectancy [23].

The mechanisms whereby disadvantage influences cardiovascular disease are complex and may include: social relationships; access to medical resources; access to healthy or unhealthy foods; harmful behavior such as smoking; inadequate or inappropriate diet; and polluted atmospheric conditions.

### Other important predictors and potential confounding

In statistical studies of exposure-response associations we need to control for other important predictors, which are unrelated to the exposure of interest, but explain some variability in the outcome and so reduce the standard errors on the estimated parameter. It is also important to assess the potential for confounding by variables that might be associated with both the exposure of interest and the health outcomes. An example that is relevant to the city of Canberra is the location of medical and aged care facilities (including aged persons’ independent living units, assisted living and high nursing home care). For instance Bynum et al found that there were fewer hospitalizations when primary care was highly integrated into a retirement community [24]. It is also possible the socio-economic disadvantage indicators of the areas in which these facilities are situated may be influenced by the location of these, which has potential to confound the statistical association between CVD and disadvantage.

In this study we explore the impact of the scale of spatial aggregation when describing the spatial distribution of selected hospital admissions for CVD and examine the associations socio-economic disadvantage has with these health outcomes.

## Methods

### Hospital admissions

Canberra is Australia’s capital city, in the Australian Capital Territory (ACT), with a population of around 380,000 people [25]. Hospital admissions for acute care of CVD for residents of Canberra between 1 January 2010 and 31 December 2012 were used from the Admitted Patient Care Data (APCD) collection. This period was chosen to match up to the population denominator data from the 2011 Census of Population and Housing conducted by the Australian Bureau of Statistics (ABS). The APCD database is maintained by the ACT Government Health Directorate and records the date of admission, primary diagnosis for each episode of care, type of care for each episode, age, sex and street address of residence for all admissions to public hospitals.

All episodes of admission to acute care were selected from the database. Acute care episodes are any cases where the patient is admitted (or transferred to) the hospital with the intent to cure the condition or alleviate symptoms. CVD episodes were selected based on diagnosis data which uses the 10th Revision of the International Classification of Diseases Australian Modification (ICD-10-AM) system. We used the following two groupings: Primary diagnosis of Myocardial Infarction (PMI) (ICD10 I21) and Primary diagnosis for Cardiovascular Disease (PCVD) where the primary diagnosis was any of the circulatory diseases (ICD10 I00-I99). This is consistent with the terminology used by the WHO Global Health Estimates project (http://terrance.who.int/mediacentre/data/ghe/). PMI occurs when blood flow stops to a part of the heart, and is commonly known as a ‘heart attack’.

To avoid artefactual inflation of admission counts by a few high-frequency patients we excluded readmission episodes from the total counts of PMI if a subsequent admission for myocardial infarction occurred within the same month of a prior admission for any person. Re-admissions were also excluded from the total count of PCVD if the person was admitted to hospital within the same month with any CVD code as the primary diagnosis. Therefore each person could only be counted once per month as an admission with primary diagnosis of PMI or PCVD.

The hospital database was geocoded for the patient’s residential address by the ACT Government and released at SA1 level. Ethical approval was obtained (ACT Health Ethics Protocol: ETH.11.14.310 and the University of Canberra Ethics Protocol: 12-158).

### Spatial aggregation at varying scales

#### Statistical Area Level 1

The Statistical Area Level 1 (SA1) is one level larger than the smallest census unit in the census boundaries of the Australian Statistical Geography Standard (ASGS) [26]. The smallest unit is called a Meshblock (there is a very limited set of non-identifiable data published at that scale, such as total residents and land use class). Our study SA1s contained between 110 to 1711 persons. There were 848 SA1s with a median area of 0.20 square km (range = 0.01 to 3.64), covering a total area of 257 square kilometers in southeastern Australia.

#### Statistical Area Level 2

Statistical Area Level 2 (SA2s) are the next level up from SA1 in the hierarchical set of spatial units of Australian census geography. SA2s generally represent an equivalent size to an Australian Suburb (e.g. a distinct neighborhood of the city) [26]. Our study SA2s contained between 344 to 15873 persons. There were 88 SA2s with a median area of 2.47 square km (range = 1.09 to 11.34), with total extent of 257 square kilometers.

### Index of relative socio-economic disadvantage

The SA1 and SA2 of residence was used to assign an area-level measure of socio-economic disadvantage using the ABS socioeconomic indexes for areas (SEIFA) index of relative socio-economic disadvantage (IRSD) [27]. The IRSD is a composite measure of disadvantage indicators, and consists of variables pertaining to income, education, employment and occupation (see S1 File for more information) [28]. We standardised the original data to Z-scores based on the fact that SA1 values are known to have a national mean of 1,000 and standard deviation of 100. As the principal components used to construct the index are arbitrary with respect to their sign (positive or negative), we rescaled the index to improve intuitive interpretation. That is, we gave more-disadvantaged areas positive scores, and less-disadvantage areas negative scores. At the broader spatial level the SA2 scores were constructed from the population weighted average of SA1 level scores [28], and these were then standardised. Therefore the SA2 level area indexes were standardised around the national SA2 mean of 999 and standard deviation of 82.

### Population denominators

The resident populations for each area at the 2011 census population were obtained from the Australian Bureau of Statistics. The populations at SA1 level came from the Census TableBuilder database and represent the count of persons usually resident in each area, by 5 year age groups [29]. The SA2 level populations are the estimated resident population by 5 year age groups for the areas at each year from the ABS model of population growth and decline (Estimated Resident Populations 2001–2013, ABS Catalogue Number 3235.0).

### Age-sex standardized rates

The outcome measure was age-sex-standardized incidence rates. We used the indirect standardization method. The rate ratio is the number of incident cases observed in an area (*O*_*i*_) divided by an expected count (*E*_*i*_). Indirect adjustment uses externally specified age-sex-specific rates found from a ‘standard population’. In our case *E*_*i*_ is estimated by multiplying the age-sex specific population years-lived for each age/sex group (signified by the subscript _*jk*_) in each small area (signified by subscript _*i*_) by the overall incidence rate in that age-sex group for the entire population of Canberra 2010–2012.

$$\begin{array}{c} E_{ijk}=Pop_{ijk}\times StandardRate_{jk} \end{array}$$ (1)

The standardised incidence rate ratio is then

$$\begin{array}{c} RateRatio_{i}=\frac{\sum O_{ijk}}{\sum E_{ijk}} \end{array}$$ (2)

To account for the small numbers of hospitalization in some study populations we used Empirical Bayes (EB) rate shrinkage to down-weight some standardized rate ratios dependent on the weights estimated using Marshall’s global EB method from the ‘spdep’ R package [30]. Further information is in S1 File. When these were multiplied by the crude rate of the standard population this gives the indirectly age-sex-standardized rates (per 1000 person-years-lived) [19].

### Medical and aged care facilities

To adjust for important nuisance parameters and potential confounding we included locations of hospitals and aged care facilities. We classified small areas (both SA1 and SA2) as including a medical facility if any of the component ABS Meshblocks within them was classed as a ‘Hospital/Medical’ type [26].

Similarly, to identify areas which contain aged care facilities we used the Community Facility Information System data provided by the ACT Government Environment and Sustainable Development Directorate. Any areas that contained a building with the class ‘Aged care’ were identified.

### Statistical analysis

Analyses were performed using R statistical language and environment version 3.2.5 (http://www.r-project.org). We used penalized regression splines in Generalized Additive Models (GAMs). We used the generalized cross-validation tool in the ‘mgcv’ package of R to automatically estimate the appropriate curvature of these response functions [31]. The estimated optimal smooth on the disadvantage term was derived from a single variable model, we then used these in a secondary model to test the impact of controlling for potential confounding due to the location of aged care or medical facilities, and residual spatial autocorrelation. Further information is in the Additional file 1.

## Results

### Descriptive statistics

**Fig 1:**
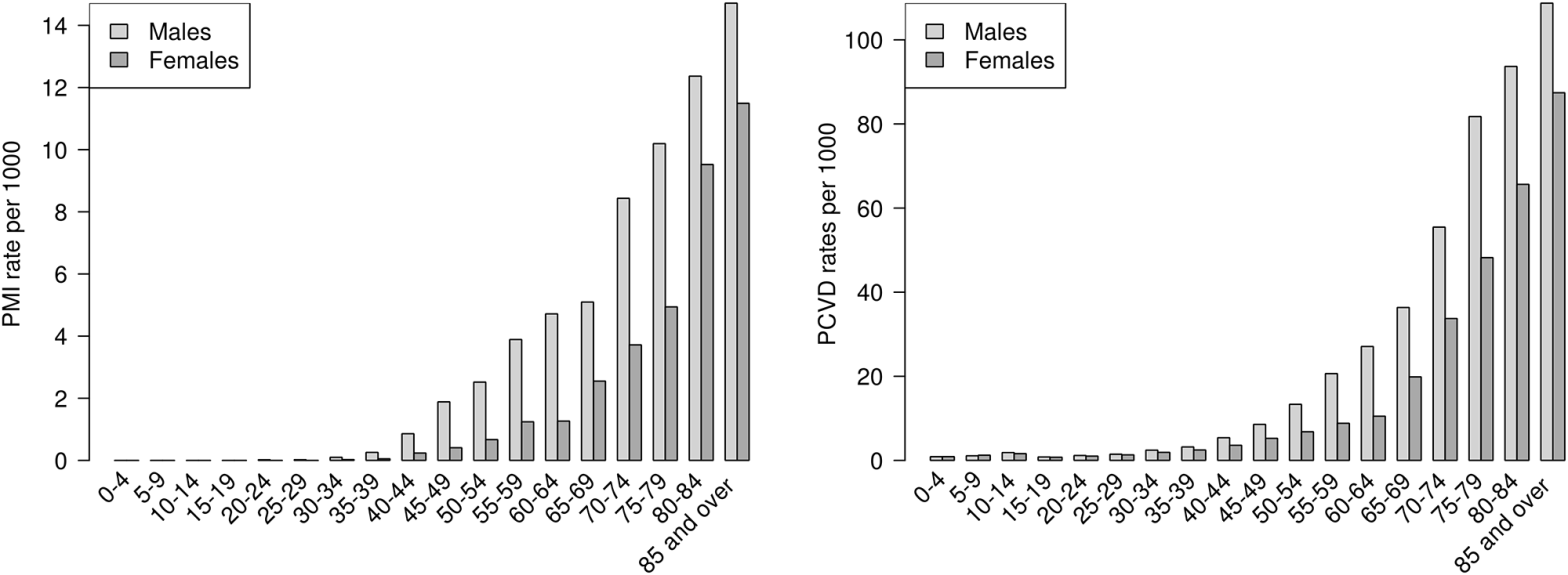
Age-specific rates by sex for a) Primary diagnosis of Myocardial Infarction (PMI) and b) Primary diagnosis of Cardiovascular Disease (PCVD) admissions in Canberra, 2010–2012.

Between 2011 and 2013, there were a total of 1,365 admissions for PMI and 10,441 hospitalizations for any PCVD code (Table 1). The rate of admissions for both groups started to increase from the ages of 40–45 and was highest for those over age 80. Hospitalization rates were consistently higher for men than women across all ages, with men having a notably larger number of admissions than women in the age groups between 45 and 79 years old (Fig 1).

**Table 1:** Total hospitalization episodes and populations (person-years-lived) for males and females, Canberra, 2010–2012.

	Females			Males			Total		
Age group	PMI	PCVD	Population	PMI	PCVD	Population	PMI	PCVD	Population
0–4	0	29	32593	0	31	35243	0	60	67836
5–9	0	37	29923	0	35	31893	0	72	61816
10–14	0	48	29839	0	57	30663	0	105	60502
15–19	0	26	34931	0	30	37044	0	56	71975
20–24	0	48	46915	1	57	48800	1	105	95715
25–29	0	60	45538	1	69	47019	1	129	92557
30–34	1	77	40253	4	98	40373	5	175	80626
35–39	2	97	39219	10	124	38785	12	221	78004
40–44	9	137	38137	32	201	37300	41	338	75437
45–49	15	193	36818	66	300	35039	81	493	71857
50–54	24	244	35832	85	450	33739	109	694	69571
55–59	39	277	31394	115	610	29561	154	887	60955
60–64	35	291	27676	125	718	26507	160	1009	54183
65–69	51	397	19993	96	685	18841	147	1082	38834
70–74	53	481	14258	111	730	13160	164	1211	27418
75–79	54	527	10931	93	746	9123	147	1273	20054
80–84	84	579	8821	80	606	6469	164	1185	15290
85 and over	107	814	9311	72	532	4893	179	1346	14204
Total	474	4362	532382	891	6079	524452	1365	10441	1056834

PMI = Primary diagnosis of Myocardial Infarction, PCVD = Primary diagnosis of Cardiovascular Disease

In our selected study region there were approximately 350,000 resident persons followed for three years, giving 1,056,834 person-years of followup. In the whole study population there was a crude incidence rate for PMI of 1.3 per 1000 person-years and 9.9 admissions per 1000 person-years for any PCVD cases. Additional descriptive statistics are in S1 File.

### Spatial patterns

#### Primary diagnosis of Myocardial Infarction (PMI)

**Fig 2:**
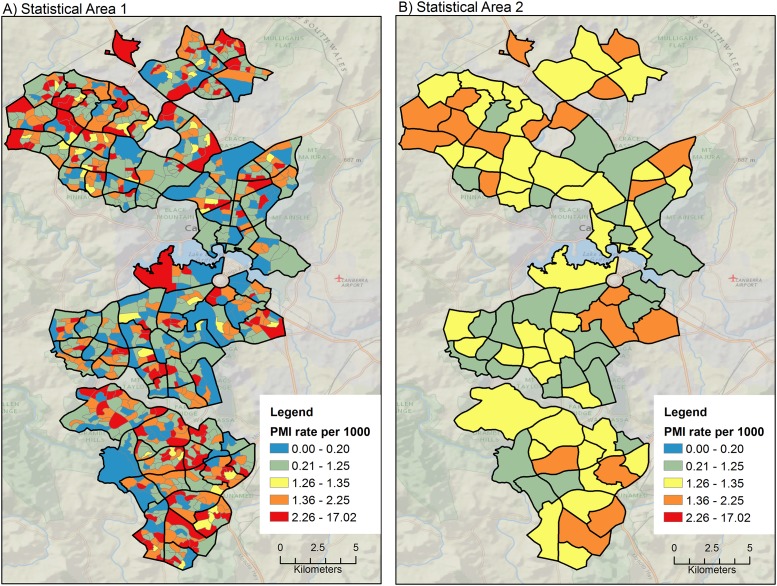
Rates of Primary diagnosis of Myocardial Infarction (PMI) in SA1 (N = 848) and SA2 (N = 88). In Panel A additional boundaries are shown for SA2 (black lines) to enable comparisons with Panel B.

The standardized incidence rate for PMI varied between 0.05 and 17.02 per 1000 person years across SA1s. Variation captured at the SA2 level of aggregation was much less (1.12–1.43). The SA2s are usually large areas that tend to ‘smooth’ the detail of the social and demographic data they portray. This effect can be seen in Fig 2 where the age-sex-standardized rates are aggregated to the SA1 and SA2 levels. The black outlines in both maps in Fig 2 are SA2, and were added to facilitate visual linking between the two maps. The broad regional trend is picked up by the SA2s and we can see that the north-western and far south areas have generally higher rates than the inner south areas. However the SA1s show there is some fine detail pattern of high rates between the central east and the southeast, and some heterogeneity in the north that is not displayed by the SA2s.

#### Primary diagnosis of Cardiovascular Disease (PCVD)

**Fig 3:**
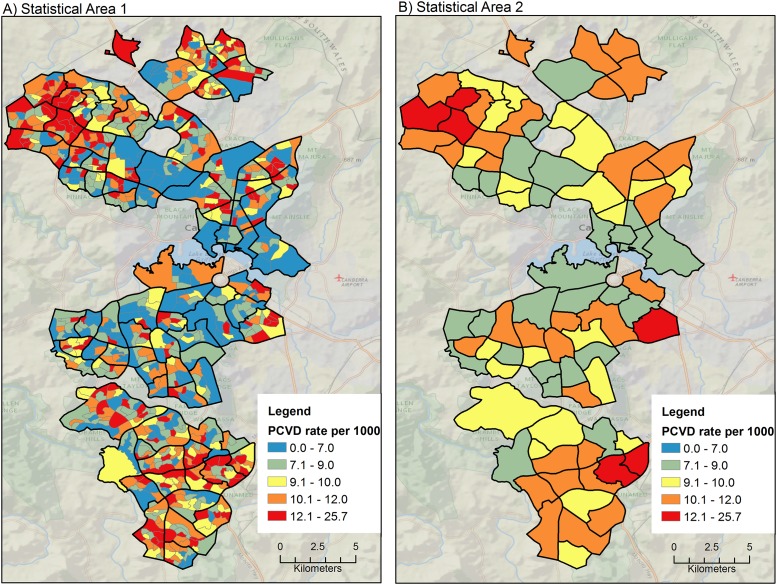
Rates of Primary diagnosis of Cardiovascular Disease (PCVD) in SA1 and SA2. In Panel A additional boundaries are shown for SA2 (black lines) to enable comparisons with Panel B.

Rates for PCVD at SA1 level varied between 3.2–25.7 per 1000 person years, whereas at the lower resolution SA2 level estimated rates varied between 7.2–13.1 per 1000 person years. The SA2 rates for PCVD shown in Fig 3 display the age-sex-standardized rates aggregated to the SA1 and SA2 levels. The broad regional trend is high in the north-western and inner-north. We can see that in the north-west there is a particular cluster of higher rates, especially when compared with the inner-south. The SA1s show this fine detail pattern of high rates most clearly. The far-south is higher than the inner south, and overall there is more heterogeneity displayed by SA1s than by the SA2s.

### Statistical associations

**Fig 4:**
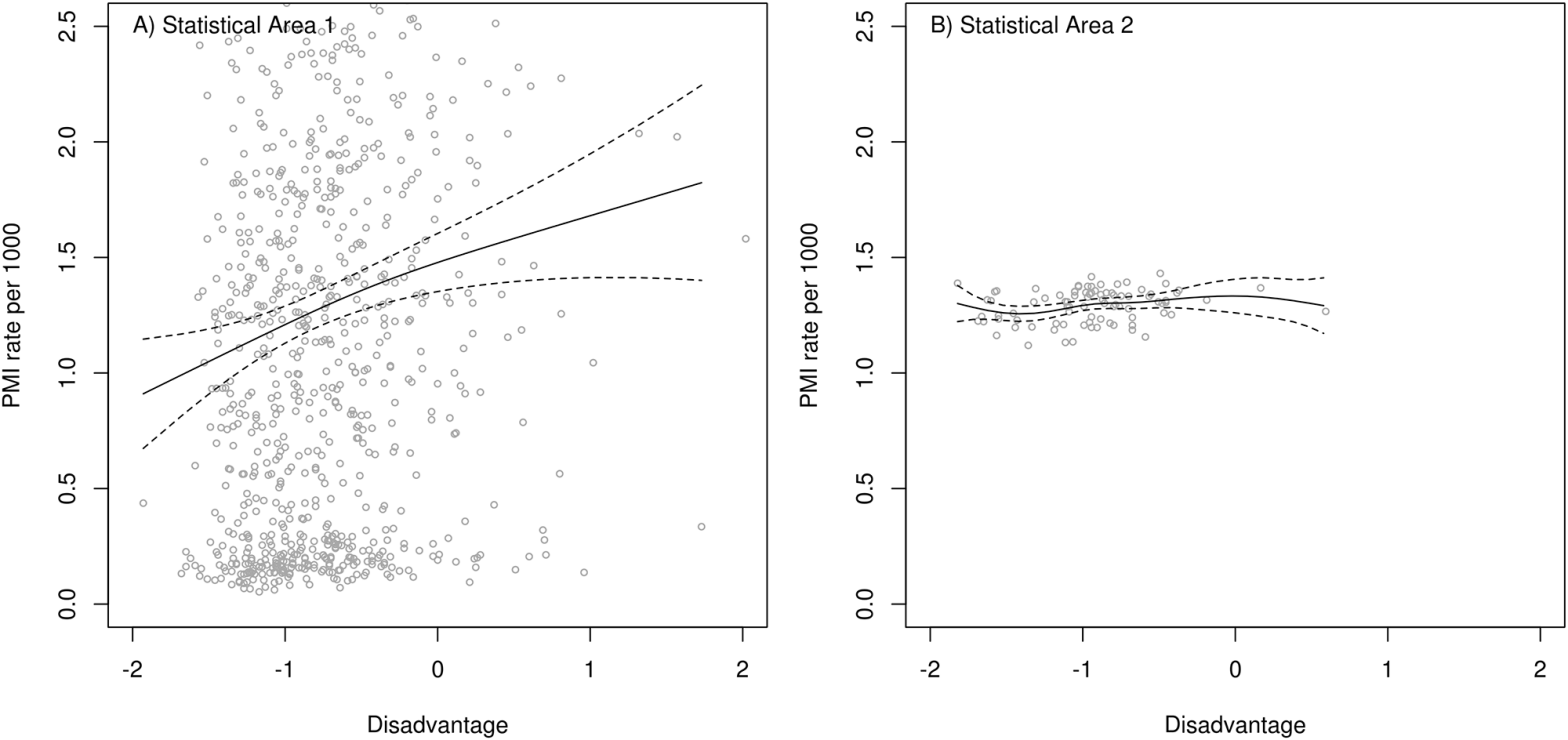
Fitted terms (solid line) and 95% confidence (dashed lines). Disadvantage increases to the right-hand side of the graph. The association between age-sex standardized rates of admissions for Primary diagnosis of Myocardial Infarction (PMI) and Disadvantage Z-scores are shown at the scale of A) Statistical Area 1 (Note: axes have been truncated to focus on the data) and B) Statistical Area 2.

**Fig 5:**
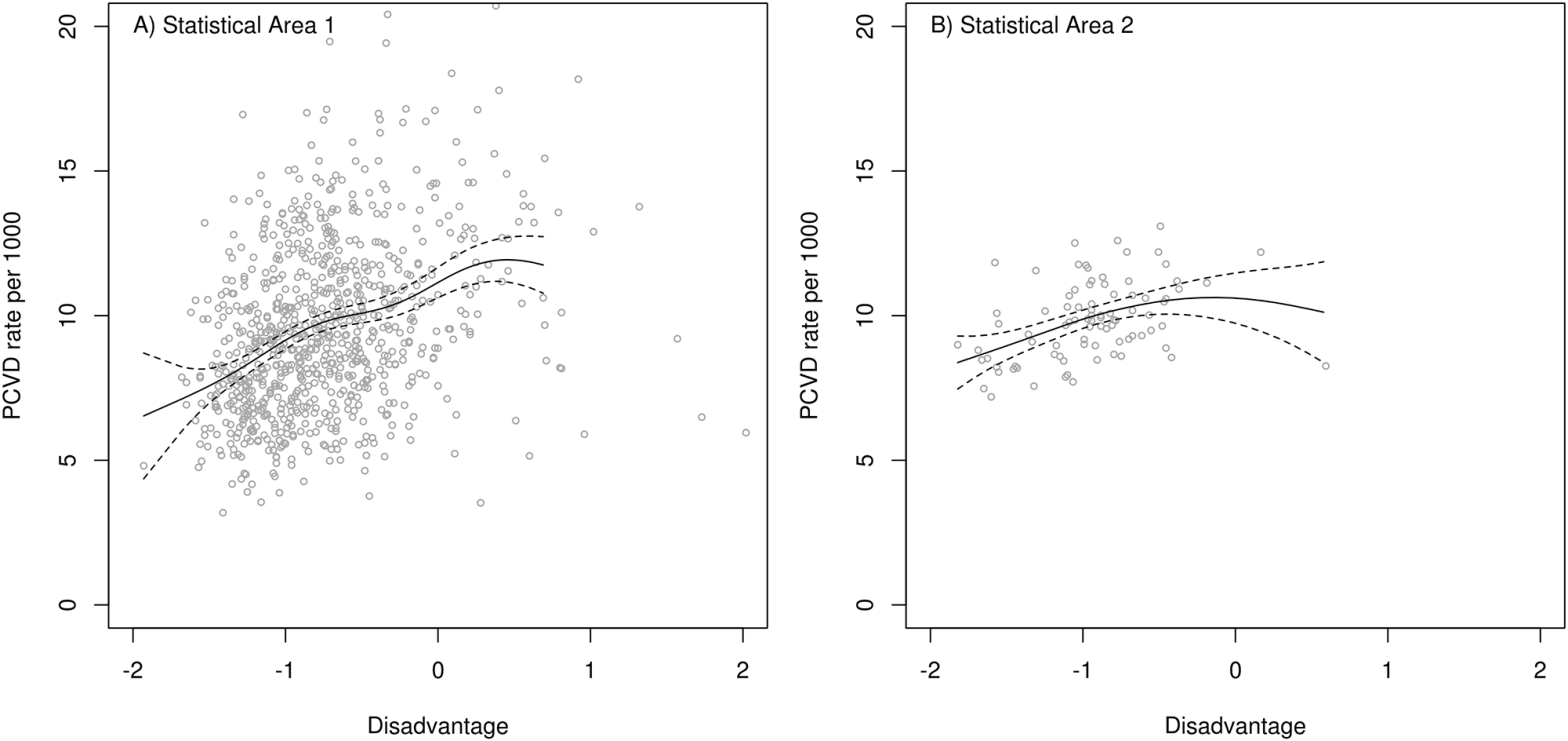
Fitted terms (solid line) and 95% confidence (dashed lines). Disadvantage increases to the right-hand side of the graph. The association between age-sex standardized rates of admissions for Primary diagnosis of any Cardiovascular Disease (PCVD) and Disadvantage Z-Scores are shown at the scale of A) Statistical Area 1 (Note: axes have been truncated to focus on the data) and B) Statistical Area 2.

The SA1 level analysis identified an increasing relative risk of hospitalization for PMI with increasing disadvantage, whereas the SA2 analysis does not (Fig 4). Fig 5 shows a similar pattern wherein the SA1 level indicates an association but the SA2 level showed a curvilinear form with no evidence that rates increased with higher disadvantage beyond the midrange.

## Discussion

### Comparison of rates across spatial scales

The rate of hospitalization for PMI and PCVD varied strongly at the SA1 level, but not at the larger level (SA2). It can be seen from Figs 2 and 3 that the spatial distribution of hospitalization rates was captured at the SA1 level of aggregation, implying that this level of geography may be better for targeted planning and resource allocation purposes. Rate variation was not captured as well when data were aggregated at SA2 level. This finding has clear implications for effective targeting of resources and public health interventions. Furthermore, it challenges the current practice of using even larger districts than SA2s for planning and allocation of public health resources (e.g. the ABS Statistical Areas level 3—SA3).

### Assessing the impact of socioeconomic disadvantage

When aggregation was considered at SA2 level, the association of rates of hospitalization for PCVD with socioeconomic disadvantage only captured a socioeconomic gradient below the middle range of disadvantage, where areas with lower disadvantage had lower rates of hospitalization. In contrast, when the exposure-response association was considered at the SA1 level there appears to be greater effect (e.g. slope). In addition the SA1 level curve increases as the levels of disadvantage increase above the mid-range, whereas at SA2 level the curve is virtually level for PMI (Fig 4) and plateaus for PCVD rates (Fig 5). The greater scatter of data points shown in both plots for SA1 give better indication of the ‘true’ picture (e.g. the drivers are multifactorial and create a wide range of health outcomes in small areas, but the socio-economic disadvantage is an important variable).

### Increased variation captured at higher spatial resolution

It can be seen from Figs 3 and 5 that the variation captured at the SA1 level is much higher than that at the SA2 level. Some of this increased variance will undoubtedly be ‘noise’ introduced by random events that are more influential on rate calculations where the underlying population denominators are small. Most of the random noise due to high variance/small numbers is smoothed through spatio-temporal aggregation and Bayesian rate shrinkage. On the other hand, much of this variation is true signal variation related to the exposure to the distribution of the socio-economic risk factors, or to other important risk factors (which may be unrelated to the factor under study). So long as these are not confounders on the putative risk factor of study (i.e. disadvantage), then including these covariates may improve model fit but will not necessarily help the interpretation of the model parameters.

Another factor that is important, from the statistical modelling perspective, is the loss of statistical power associated with a smaller number of spatial units for inclusion in the analysis at the SA2 level. Thus, despite the presence of added variance (possibly noise) at the smaller scale, the increased signal from a ‘truer’ representation of the exposure measures, and increased power from a greater number of sampling units, combine to give a better overall representation of the available data.

### Improving population health

On the basis of the research reported here for cardiovascular diseases, which are highly prevalent in this community, descriptive analysis and reporting should be carried out at the SA2 level since this captures the broad distribution of disease rates quite well. However, for explanatory or inferential studies it may then be necessary to drill down to smaller level to delineate those areas for specific focus, either to understand the likely exposures or to target resources for intervention.

Health protection and reducing health inequalities should be seen as linked measurement and policy issues. High spatial resolution analysis is essential to uncover local risk-related behaviors and exposures and to enable effective targeting of resources and monitoring of the impact of interventions. Since only limited research to date has been conducted in Australia using robust small area analyses, little is known on how levels of (and differentials in) health outcomes at the very local level of geography track over time or how they may be modified by targeted resources or public health policy changes or local health system performance.

Reductions in cardiovascular disease mortality and morbidity in developed nations have been attributed to changes in behaviors at the population level leading to better prevention and better treatments [32, 33]. However, there is evidence that these gains have not been shared equitably across populations. Differences in socioeconomic circumstances involving factors such as psychosocial risks, the physical environment, lifestyle choices, commercial exploitation, access to and quality of health care, stress and poor living and working environments may themselves cause poor health. If further gains are to be made in reducing the impact of CVD, and other non-communicable diseases, on population health, social and economic differences will need to be addressed alongside more effective dietary, lifestyle and healthcare interventions. In addition to these challenges, service delivery is becoming increasingly fragmented in many developed nations with the privatization of many aspects of service delivery as well as devolving many public health responsibilities to local governments. Under such a scenario, it is even more important that key decisions are driven by (and their effects are monitored by) rigorous comparative analysis of health outcomes at high spatial resolution (small area) to ensure that resources are allocated effectively and fairly to benefit public health [34, 35].

### Strengths and limitations

#### Strengths

Most importantly, the high spatial resolution of the data was a key strength of this mapping and modelling study. In addition our model fitting used Bayesian rate shrinkage to derive robust small-area incidence rate estimates. These methods give the best available account of age-sex group differences at such small scale.

#### Limitations

A key limitation of this study is the potential cross-level fallacy (e.g. the so-called ‘ecological’ fallacy), but this also applies to all aggregate-level data modelling. This is the problem of using associations between area-level indicators of disadvantage and cardiovascular disease rates to infer associations at individual level. This weakness is unavoidable in this study design, but the risks from this can be mitigated by maintaining a conservative attitude to inferences, and assessing the consistency of findings with other studies, the coherence of findings with the generally known facts of the ecology of disease, the plausibility of the mechanisms postulated, and assessment of a dose-response gradient (along with the other elements of Bradford Hill’s criteria for causal inference [36]).

In our study we applied a conservative and cautious statistical modelling approach using penalized regression to estimate the area level relationships. We readily acknowledge that furthering our understanding would need to assess the importance of individual level processes including exposure and susceptibility. The individual level however is not available for analysis using these administrative data, given the confidentiality concerns currently restricting access to high resolution data. Because of this, aggregate-level data is often used in spatial epidemiology to describe the geographical contexts for studies of morbidity rates and potential drivers.

Another limitation is that the effects of in- and out- migration, and daily movements between areas is not captured precisely in the data available, which may lead to misclassification and error in rate calculations. However, the 2011 Census statistics for the SA1s in our analysis show that the majority of residents had not changed address from 2010 (with the SA1 mean of 82% of people with same place of residence).

Such small-area analysis is only feasible where exposures and event rates are sufficiently high to be detectable within small geographical areas over relatively short time frames (e.g. limited person years of follow up). Again, there is limited individual level data available to support alternative analyses.

Furthermore, the analysis made use only of public hospital data because that was all that were available. However, it is likely that public hospitals account for only 60% of admissions on average across the population, based on the statistics from other jurisdictions [37]. The Australian Institute of Health and Welfare have found that in 2009–2010 the ratio of hospitalizations for patients living in disadvantaged areas of public hospitals to private hospitals was 2.85 (291 per 1000 compared to 102 per 1000 respectively), whereas in the least disadvantaged areas the ratio of residents who utilized public hospitals to those using private hospitals was 1.51 (rates of 221 per 1000 compared with 146 per 1000 respectively) [37]. Therefore this bias is less likely to influence the hospital utilization patterns in the most disadvantaged areas, and our finding of increasing relative risks associated with increasing socio-economic disadvantage above the midrange (shown by our SA1 level analysis) may not be affected. But further research is required to ascertain how robust these findings are.

## Conclusion

The choice of scale of aggregation is critically important in the spatial epidemiology of highly prevalent non-communicable diseases such as CVD. It is notable that the exposure-response association for PCVD at the SA1 level is somewhat curved with a slight plateau at high disadvantage. However it is also apparent that, compared to the SA2 data, there are many SA1s with high disadvantage scores (above the national average). The fact that the curve does continue upward from the mid-range in the SA1 analysis provides more compelling evidence that there is high risk of CVD hospitalization related to socio-economic disadvantage in Canberra.

When data were aggregated at the high spatial resolution SA1 level, estimated age-sex standardized rates of any primary diagnosis CVD hospital admissions across the small areas varied much more than when they were aggregated at SA2. This suggests two things: 1) the primary drivers of CVD may be highly local i.e. in close proximity to individual residences in the most exposed areas and 2) aggregation to a larger geographic scale may smooth localized variation within the larger area, effectively attenuating ‘signal’ in the data.

This loss of signal becomes vitally important when one is interested in identifying or determining the role of potential explanatory factors in the development of non-communicable diseases. In this study we have examined the relationship of rates of hospital admissions for CVD with socio-economic disadvantage. Effective loss of signal (loss of statistical power) when aggregating at SA2 level meant that we were barely able to detect any potential influence of disadvantage on hospitalizations for myocardial infarction and only effects of low to moderate disadvantage on hospital admissions rates due to any cardiovascular disease. In contrast, when data were aggregated at the smaller SA1 level, the influence of disadvantage could be detected across the full range. If true, and replicated elsewhere, these findings have major implications for the scale of data collection for surveillance of highly prevalent non-communicable diseases.

## Supporting information

S1 File Supporting information document for the article. Description of datasets, model selection, regression diagnostics and sensitivity assessments.
(PDF)
