# Supplementary material for: Impact of scale of aggregation on associations of cardiovascular hospitalization and socio-economic disadvantage
Source: PLoS One. 2017 Nov 28;12(11):e0188161. doi: 10.1371/journal.pone.0188161 (PMC5705072; doi:10.1371/journal.pone.0188161)
Supplement: S1 File — Description of datasets, model selection, regression diagnostics and sensitivity assessments. (PDF) [file pone.0188161.s001.pdf]

Supporting Information document for the article: 'Impact of scale of aggregation on associations of cardiovascular hospitalization and socio-economic disadvantage'

Ivan C. Hanigan * [1,2] Thomas Cochrane [1] Rachel Davey [1]

[1] *Spatial Epidemiology Group, University of Canberra, Canberra, Australia*

[2] *University of Sydney, Sydney, Australia*

* *Corresponding author: ivan.hanigan@canberra.edu.au*

Compiled from Rmarkdown on September 13, 2017

Contents

Indirect standardization and Empirical Bayes rate shrinkage 2

Penalized regression splines 2

Index of Relative Socio-Economic Disadvantage (IRSD) 3

Descriptive statistics 4

Principal diagnosis of any Cardiovascular Disease (PCVD) 5
- PCVD SA1 . . . 5
- PCVD SA2 . . . 6
- Assessment of residual spatial autocorrelation using semi-variograms . . . 7
- SA1 global Moran's I tests for residual spatial autocorrelation . . . 8
- SA1 local indicators of spatial association in residuals . . . 9
- SA2 global Moran's I tests for residual spatial autocorrelation . . . 10
- SA2 local indicators of spatial association in residuals . . . 11
- PCVD models at SA1 and SA2: Sensitivity of regression to residual autocorrelation . . . 12

Principal diagnosis of Myocardial Infarction (PMI) 13
- PMI SA1 . . . 13
- PMI SA2 . . . 14
- Assessment of residual spatial autocorrelation using semi-variograms . . . 15
- SA1 global test of residual spatial autocorrelation . . . 16
- SA1 local indicators of spatial association in residuals . . . 17
- SA2 global test of residual spatial autocorrelation . . . 18
- SA2 local indicators of spatial association in residuals . . . 19
- PMI SA1 vs SA2 . . . 20

Reproducibility statement 21

References 22

Indirect standardization and Empirical Bayes rate shrinkage

We used indirect standardization to adjust for different age and sex structure in our study populations: the ratio of Observed counts of disease O_i to Expected counts E_i (derived from an externally specified standard population). Although this method is potentially more biased with heterogeneous age-sex structures, it is more precise and so appropriate than direct standardization when populations are very small as is our case (Meade & Earickson 2000). However, in study populations where the number of people in each age-sex group can be very small, even the indirect method can result in unstable estimates of the incidence rate ratios. This is because the age-sex specific rates of the standard population are based on much larger numbers than those of the study populations, which may have observed numbers of events that fluctuate wildly. To address this potential problem we used Marshall's global Empirical Bayes estimator in the R package `spdep` to shrink the estimated incidence rate ratios toward the global mean (Bivand *et al.* 2008). The shrinkage highly depends on the value of E_i. If it is large then the rate ratio is considered a reliable estimate, and the estimator will give more weight to it. Conversely, if E_i is small, more weight is given to the prior estimate (the mean of all study population rate ratios) because the rate ratio is less reliable and the shrinkage is calculated based on the prior mean and variance and EB shrunken incidence rates ratios are the result (Bivand *et al.* 2008).

Penalized regression splines

We used penalized regression splines in Generalized Additive Models (GAMs) to model the associations between the exposure variables and the hospitalization outcome. We used the generalized cross-validation tool in the 'mgcv' package of R to automatically estimate the appropriate curvature of these response functions (Wood 2006, 2008).

To identify if there were non-linear associations, and assess the impact of controlling for potential confounding, two linear regression models were estimated for each level of spatial units (e.g. SA1 or SA2):

$$Y_i = \beta_0 + s(Disadvantage_i) \qquad \text{(Model 1)}$$

$$\begin{aligned} Y_i = \beta_0 &+ s(Disadvantage_i, knots = EDF) \\ &+ \beta_2 Aged_i + \beta_3 Medical_i \\ &+ s(x, y) \end{aligned} \qquad \text{(Model 2)}$$

Where:

Y_i is the incidence rates (weighted with global Empirical Bayes shrinkage)

$s()$ is the penalized regression spline

$knots = EDF$ is the Estimated Degrees of Freedom from Model 1

$s(x, y)$ is the penalized regression spline representation of a trend surface based on the longitude (x) and latitude (y) of the centroid for each area (population weighted centroids in the case of SA2). The penalties on the smooth are equivalent to modelled spatial random effects (Wood 2006).

Thus using the estimated optimal smooth on the disadvantage term from Model 1, we use Model 2 to test the impact of controlling for potential confounding due to the location of aged villages, medical facilities, and residual spatial autocorrelation.

Index of Relative Socio-Economic Disadvantage (IRSD)

The IRSD summarizes variables that indicate relative disadvantage at the SA1 level, according to the concept described in the ABS technical report (Australian Bureau of Statistics 2013).

The variables used in 2011 were:

- Percent people with stated annual household equalized income between $1 and $20,799 (approx. 1st and 2nd deciles);
- Percent families with children under 15 years of age who live with jobless parents;
- Percent occupied private dwellings with no internet connection;
- Percent employed people classified as 'labourers';
- Percent people aged 15 years and over whose highest level of education is Year 11 or lower. Includes Certificate I and II;
- Percent people (in the labour force) unemployed;
- Percent occupied private dwellings paying rent less than $166 per week (excluding $0 per week);
- Percent one parent families with dependent offspring only;
- Percent people aged under 70 who have a long–term health condition or disability and need assistance with core activities;
- Percent occupied private dwellings with no cars
- Percent people aged 15 and over who are separated or divorced;
- Percent occupied private dwellings requiring one or more extra bedrooms (based on Canadian National Occupancy Standard);
- Percent employed people classified as Machinery Operators and Drivers;
- Percent employed people classified as Low Skill Community and Personal Service Workers;
- Percent people aged 15 years and over who have no educational attainment; and
- Percent people who do not speak English well.

Descriptive statistics

Table 1: Selected descriptive statistics

	Statistical Area 1	Statistical Area 2
Number of areas (with disadvantage scores)	848 (833 non-missing)	88 (87 non-missing)
Median/range of disadvantage scores	-0.81 (range = -1.93 to 3.11)	-0.94 (range = -1.82 to 0.59)
Median/range of area (Sq Km)	0.20 (range = 0.01 to 3.64)	2.47 (range = 1.09 to 11.34)
Median/range of population (annualized)	392 (range = 110 to 1711)	3352 (range = 344 to 15873)
Median/range PMI (1000 person years)	1.13 (range = 0.05 to 17.02)	1.31 (range = 1.12 to 1.43)
Median/range PCVD (1000 person years)	9.18 (range = 3.19 to 25.74)	9.83 (range = 7.19 to 13.09)
Areas with aged care and disadvantage score	44	32
Areas with medical and disadvantage score	1	3

Principal diagnosis of any Cardiovascular Disease (PCVD)

PCVD SA1

The diagnostics for Model 1 for PCVD at SA1 level showed an adequate model parametrization (shown in Figure 1).

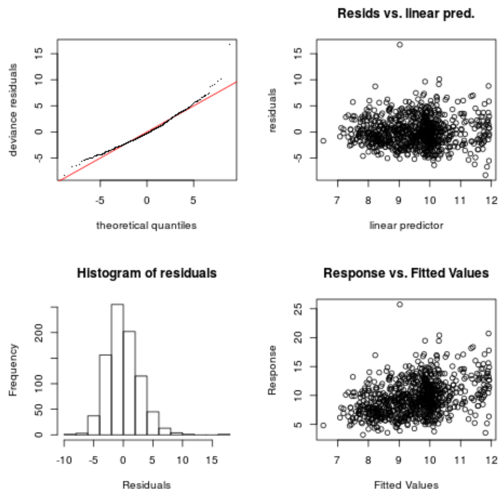

Figure 1: Diagnostics for Model 1 for PCVD at the SA1 level.

Model 1 is compared to Model 2 in Figure 2 the exposure-response curve for disadvantage from Model 1 is almost identical to the curve from Model 2. Notably SA1s that contain aged villages appear to have substantially reduced relative risk.

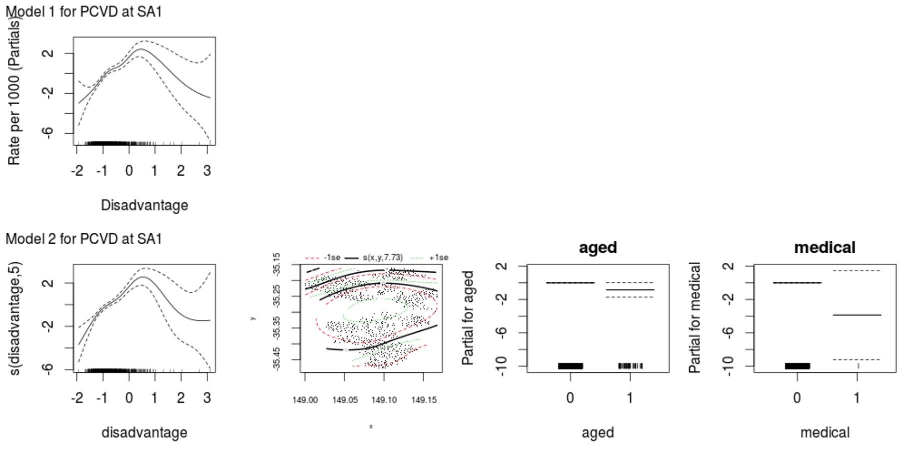

Figure 2: Comparison of predictors in Model 1 and Model 2 for PCVD at the SA1 level.

PCVD SA2

The diagnostics for Model 1 for PCVD at the SA2 level are shown in Figure 3.

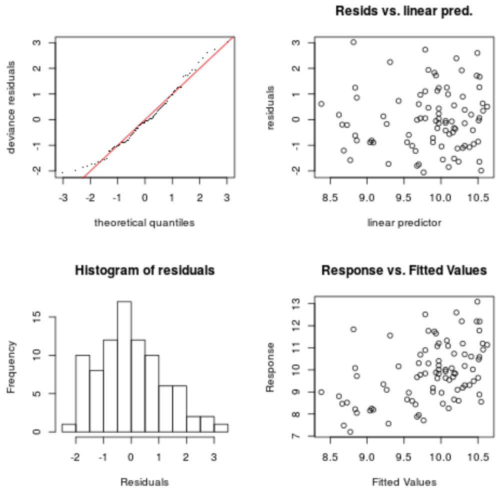

Figure 3: Diagnostics for Model 1 for PCVD at the SA2 level.

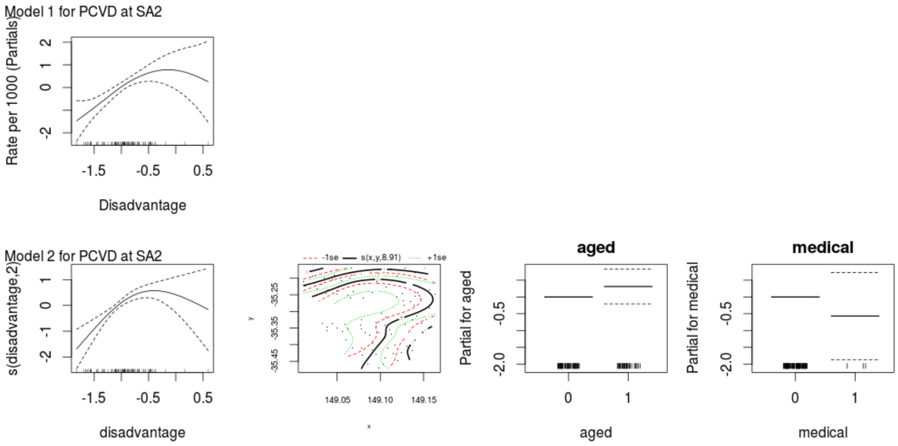

Figure 4: Comparison of predictors in Model 1 and Model 2 for PCVD at the SA2 level.

When Model 1 is compared to Model 2 in Figure 4 the exposure-response curves for disadvantage are almost identical. At the scale of SA2 the association with aged villages is in the opposite direction (indicating increased incidence rates) but this was not statistically significant. Medical facilities were not associated with PCVD in this model.

Assessment of residual spatial autocorrelation using semi-variograms

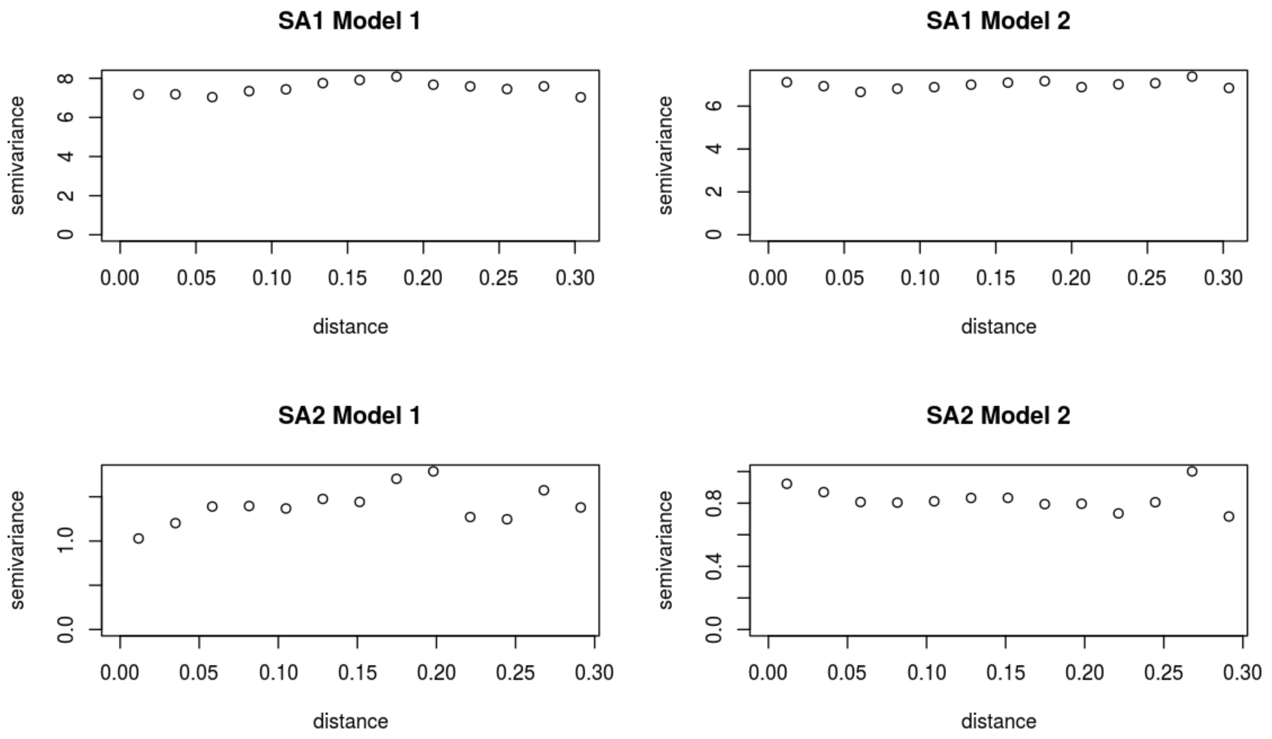

Figure 5: PCVD Model 1 and Model 2 variograms of spatial autocorrelation in the residuals.

The strong assumption of GAMs that the residuals are independent and identically distributed may be violated by the failure to account for some important spatially varying predictor, and this may result in spatial autocorrelation in the residuals which will affect the standard errors and p-values. The variogram of the residuals using the ‘geoR’ package of R can be used to examine this. The plotted variogram for uncorrelated residuals should give a more or less flat variogram, especially at the left hand side where distances are small between data points. Un-modelled spatial autocorrelation usually results in a variogram which increases sharply before eventually plateauing (Wood 2006). From the variograms in Figure 5 it appears that SA1 level models 1 and 2 both show no residual spatial autocorrelation. These plots do suggest that at SA2 level Model 1 might contain some residual spatial autocorrelation, whereas this is not evident in SA2 level Model 2.

SA1 global Moran's I tests for residual spatial autocorrelation

The semi-variogram approach to assessing spatial autocorrelation used above reflects a distance based relationship. However, in our case-study, distance is likely to be less of an issue than the spatial configuration of the city. Canberra has an interesting configuration whereby the populated areas are concentrated in 'town centers' distributed almost like islands around the civic center. These towns are then surrounded by nature reserves. Therefore, the analysis of spatial autocorrelation becomes more complicated, and issues can arise such as edge effects.

We also assessed global Moran's I tests using the `spdep` package in R (Bivand *et al.* 2008). These tests used different neighborhood representations such as the 'Queens' and 'Rook' adjacency types, at different spatial lags (measured by first-, second- or third-order neighbour status), and different distance bands (by identifying neighbours in the 0-1, 1-2 and 2-3 kilometer distance bands). Potential edge effects were anticipated and so we used a neighbor weighting scheme which up-weights observations with many neighbours.

The results shown in Table 2 demonstrate that there was indeed evidence of locally autocorrelated residuals from Model 1 (Moran's I tests with p-value less than 0.01), while Model 2 did not. This suggests that the spatial spline term in Model 2 explained most of the correlated residual deviance.

	I	E(I)	var(I)	St. deviate	p-value
PCVD, Model 1, Queen, Order 1	0.0659	-0.0012	0.0004	3.1696	0.0008
PCVD, Model 1, Queen, Order 2	0.0663	-0.0012	0.0002	4.4966	0.0000
PCVD, Model 1, Queen, Order 3	0.0607	-0.0012	0.0002	4.9149	0.0000
PCVD, Model 1, Rook, Order 1	0.0537	-0.0012	0.0005	2.4813	0.0065
PCVD, Model 1, Rook, Order 2	0.0605	-0.0012	0.0003	3.8537	0.0001
PCVD, Model 1, Rook, Order 3	0.0704	-0.0012	0.0002	5.2785	0.0000
PCVD, Model 2, Queen, Order 1	-0.0145	-0.0012	0.0004	-0.6259	0.7343
PCVD, Model 2, Queen, Order 2	-0.0050	-0.0012	0.0002	-0.2524	0.5996
PCVD, Model 2, Queen, Order 3	-0.0029	-0.0012	0.0002	-0.1362	0.5542
PCVD, Model 2, Rook, Order 1	-0.0244	-0.0012	0.0005	-1.0491	0.8529
PCVD, Model 2, Rook, Order 2	-0.0128	-0.0012	0.0003	-0.7221	0.7649
PCVD, Model 2, Rook, Order 3	0.0038	-0.0012	0.0002	0.3661	0.3572
PCVD, Model 1, Band 0-1	0.0528	-0.0012	0.0002	3.5100	0.0002
PCVD, Model 1, Band 1-2	0.0586	-0.0012	0.0001	6.1269	0.0000
PCVD, Model 1, Band 2-3	0.0299	-0.0012	0.0001	3.7057	0.0001
PCVD, Model 2, Band 0-1	-0.0205	-0.0012	0.0002	-1.2499	0.8943
PCVD, Model 2, Band 1-2	0.0003	-0.0012	0.0001	0.1529	0.4392
PCVD, Model 2, Band 2-3	-0.0191	-0.0012	0.0001	-2.1250	0.9832

Table 2: Global Moran's I tests for SA1 with different adjacency definitions, spatial lags (Order) and distance bands (kilometers)

SA1 local indicators of spatial association in residuals

To explore the local spatial autocorrelation in the residual deviance from both models we conducted a Local Indicator of Spatial Association (LISA) analysis. We used the Local Moran's I tests for first order neighborhoods (defined with the Queen adjacency method) (Bivand *et al.* 2008). Clusters were identified as areas with test scores significant at the 0.01 p-value level. In Figure 6 the red polygons highlight SA1 with high residual values surrounded by SA1s with high residuals. Blue polygons highlight SA1 with low residuals surrounded by SA1s with low residuals. These maps identify some clusters of several SA1s in the residuals from Model 1, whereas this was less evident in the residuals from Model 2, which implies that the spatial spline term did control for some (but not all) the additional spatial deviance not captured by the socio-economic disadvantage predictor. Therefore we conclude that Model 2 was strong enough to pick up both structured and unstructured spatial variability, except for a very few SA1s for which clustered high residual values are indicative of the presence of other unmeasured potential predictors of the outcome variable.

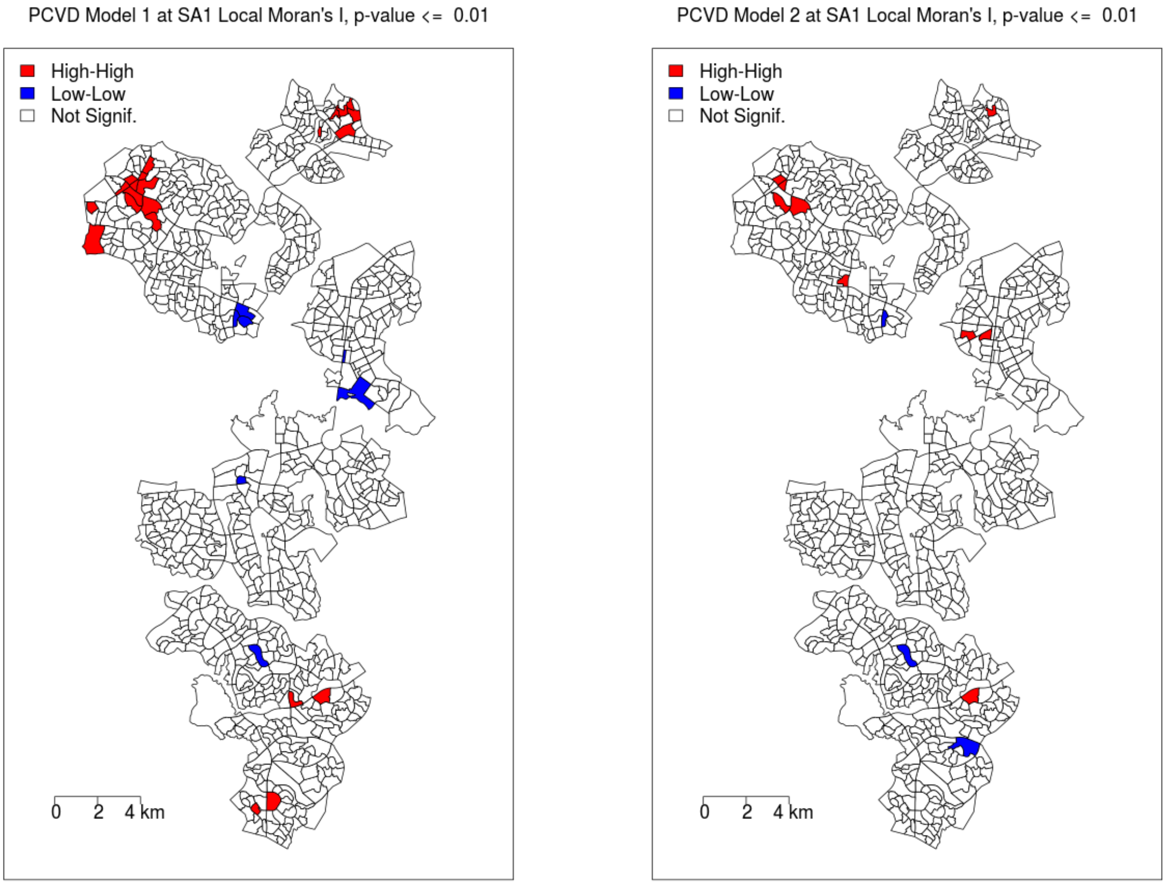

Figure 6: PCVD Model 1 and Model 2 at SA1 LISA maps of significantly High-High (red) and Low-Low (blue) clustered residuals with p-values less than or equal to 0.01 using first order Queen neighbors.

SA2 global Moran's I tests for residual spatial autocorrelation

Global Moran's I tests of the different neighborhood representations (the 'Queens' and 'Rook' adjacency types), at different spatial lags, and different distance bands (in this case identifying neighbors in the 0-2, 2-4 and 4-6 kilometer bands) are shown in Table 3. These demonstrate that there was evidence of locally autocorrelated residuals from Model 1 (Moran's I tests with p-value less than 0.01), while Model 2 did not. This suggests that the spatial spline term in Model 2 captured the correlated residual deviance.

	I	E(I)	var(I)	St. deviate	p-value
PCVD, Model 1, Queen, Order 1	0.1826	-0.0118	0.0047	2.8262	0.0024
PCVD, Model 1, Queen, Order 2	0.0322	-0.0120	0.0032	0.7772	0.2185
PCVD, Model 1, Queen, Order 3	0.0215	-0.0125	0.0035	0.5717	0.2838
PCVD, Model 1, Rook, Order 1	0.2060	-0.0118	0.0058	2.8707	0.0020
PCVD, Model 1, Rook, Order 2	0.0322	-0.0118	0.0038	0.7133	0.2378
PCVD, Model 1, Rook, Order 3	0.0167	-0.0125	0.0038	0.4761	0.3170
PCVD, Model 2, Queen, Order 1	-0.0878	-0.0118	0.0047	-1.1069	0.8658
PCVD, Model 2, Queen, Order 2	-0.1054	-0.0120	0.0032	-1.6409	0.9496
PCVD, Model 2, Queen, Order 3	0.0507	-0.0125	0.0035	1.0635	0.1438
PCVD, Model 2, Rook, Order 1	-0.0805	-0.0118	0.0057	-0.9078	0.8180
PCVD, Model 2, Rook, Order 2	-0.1371	-0.0118	0.0038	-2.0349	0.9791
PCVD, Model 2, Rook, Order 3	0.0340	-0.0125	0.0038	0.7575	0.2244
PCVD, Model 1, Band 0-2	0.1471	-0.0122	0.0074	1.8470	0.0324
PCVD, Model 1, Band 2-4	0.0958	-0.0118	0.0028	2.0419	0.0206
PCVD, Model 1, Band 4-6	0.0030	-0.0118	0.0023	0.3101	0.3782
PCVD, Model 2, Band 0-2	-0.1324	-0.0122	0.0074	-1.3957	0.9186
PCVD, Model 2, Band 2-4	-0.1002	-0.0118	0.0028	-1.6814	0.9537
PCVD, Model 2, Band 4-6	0.0214	-0.0118	0.0023	0.6993	0.2422

Table 3: Global Moran's I tests for SA2 with different adjacency definitions, spatial lags (Order) and distance bands (kilometers)

SA2 local indicators of spatial association in residuals

LISA mapping analysis at the SA2 level are shown in Figure 7. As before we used the Local Moran's I tests for first order neighborhoods defined with the Queen adjacency method. Areas with test scores significant at the 0.01 p-value level are highlighted by the red polygons (SA2 with high residual values surrounded by SA2s with high residuals). These maps identify some clusters of SA2s with positive residuals from Model 1, whereas none were evident in the residuals from Model 2, which implies that the spatial spline term controlled for any spatial deviance not captured by the predictors. There were no SA2 clusters of low residuals and hence no blue polygons.

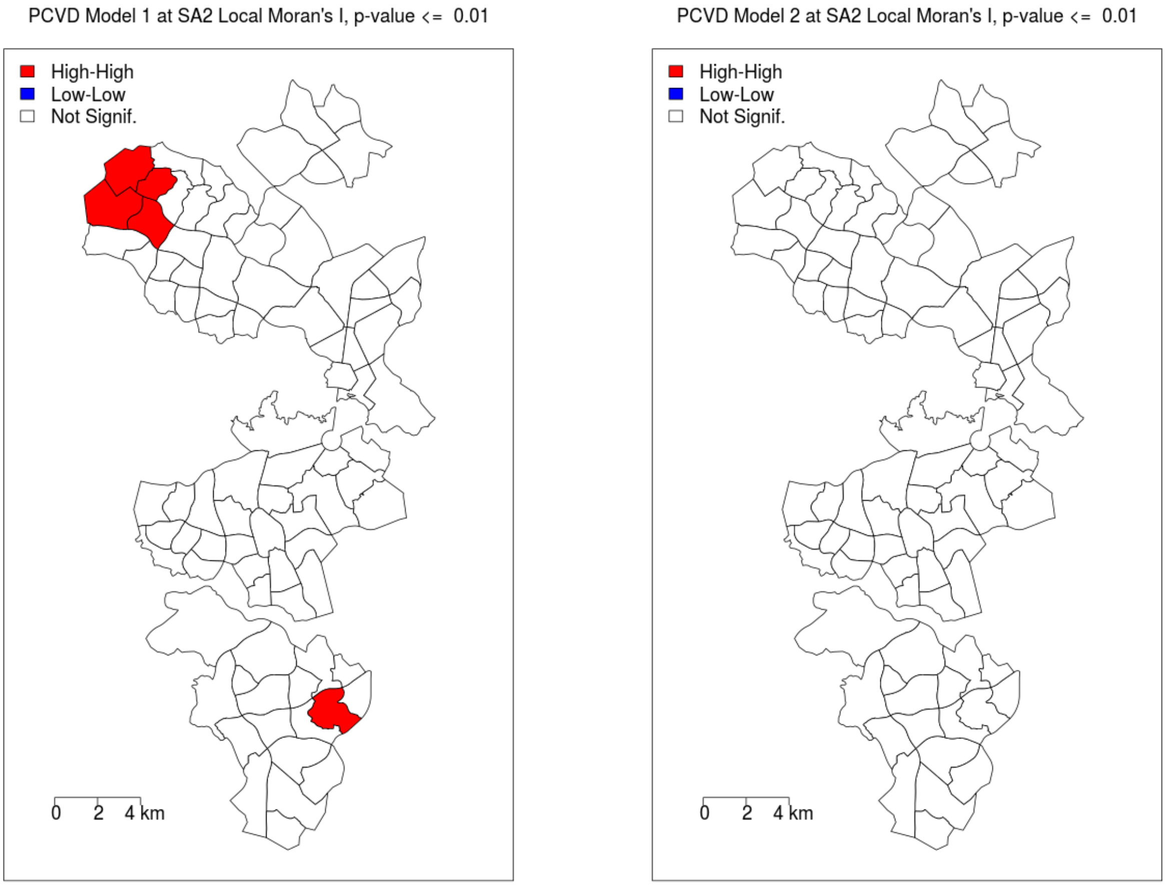

Figure 7: PCVD Model 1 and Model 2 at SA2 LISA maps of significantly High-High (red) clustered residuals with p-values less than or equal to 0.01 using first order Queen neighbors. There were no Low-Low (blue) clusters.

PCVD models at SA1 and SA2: Sensitivity of regression to residual autocorrelation

The analyses of residual spatial autocorrelation shown above do not directly address the question whether the interpretation of the association from our regression models are sensitive to the existence of correlated errors. To do this we investigated the shape of the partial smooths from the non-spatial Model 1 compared to the spatial Model 2. In Figure 8 the fitted disadvantage response curves from Model 1 and Model 2 are shown. These curves show the response function for that term given all other terms being held equal. This presentation of the results from the two models side by side suggest that the interpretation of the regression model is not changed after the additional control for spatially correlated errors in Model 2.

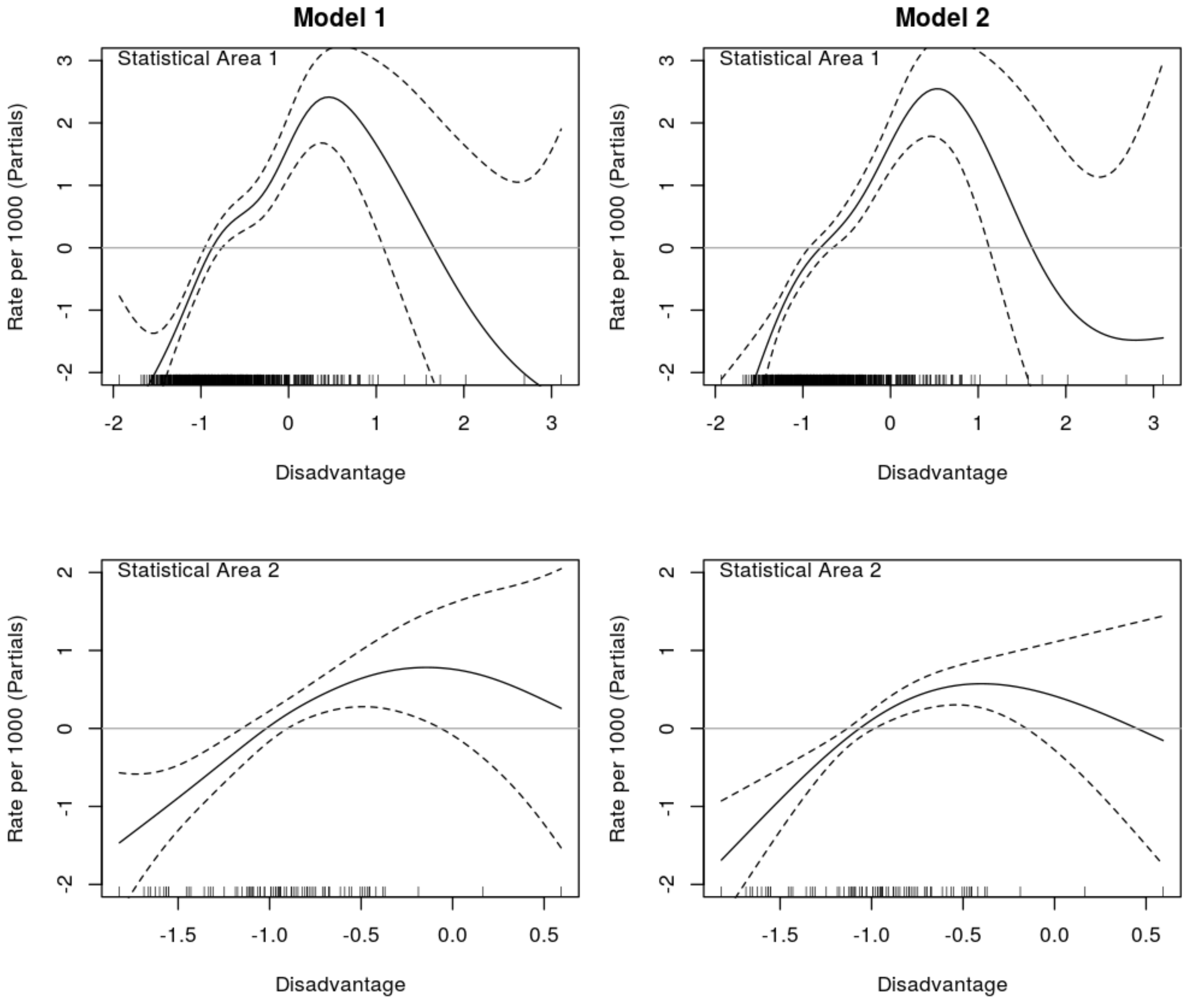

Figure 8: Comparison of Model 1 and Model 2 for PCVD at SA1 and SA2 level.

Principal diagnosis of Myocardial Infarction (PMI)

PMI SA1

In the first implementation of Model 1 for PMI at the SA1 level the model diagnostics identified a data point with an extremely large residual in the fitted model. This outlier was excluded leaving 832 data points and the resulting model summary is below. Model checking diagnostics showed an adequate model parametrization (shown in Figure 9).

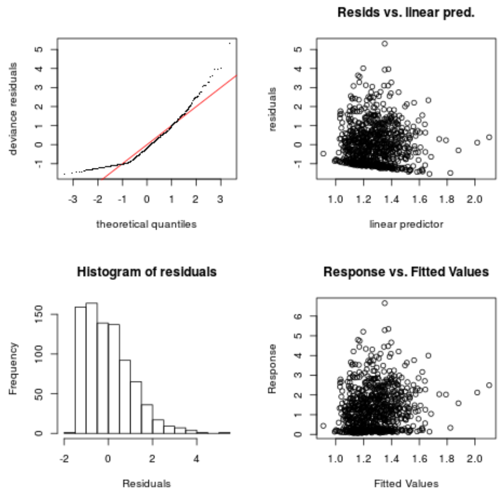

Figure 9: Diagnostics for Model 1 for PMI at the SA1 level.

Model 1 is the minimal model and so we assessed it in comparison to Model 2 which adjusted for potential confounding by aged villages, medical facilities and residual spatial auto-correlation. In Figure 10 the exposure-response curve for disadvantage from Model 1 is almost identical to the curve from Model 2. Notably SA1s that contain aged villages had reduced relative risks of hospitalization, possibly due to care and services. SA1s with medical facilities also had lower estimated relative risks, but this was not statistically significant.

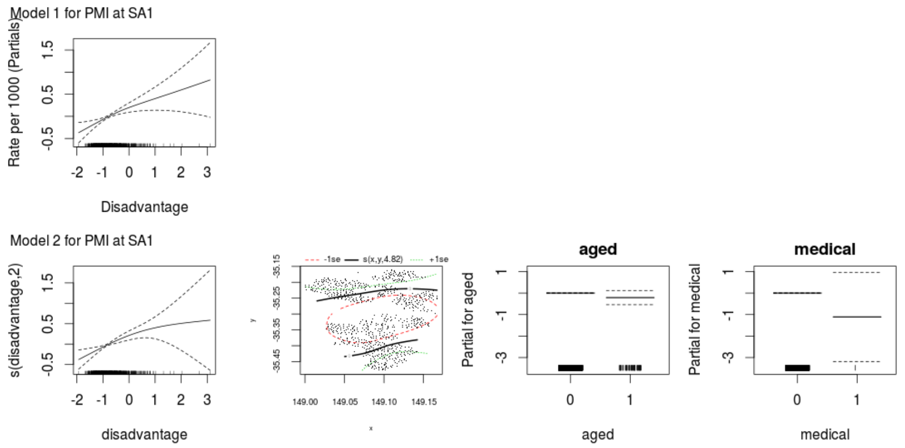

Figure 10: Comparison of predictors in Model 1 and Model 2 for PMI at the SA1 level.

PMI SA2

The model diagnostics for Model 1 for PMI at the SA2 level are shown in Figure 11.

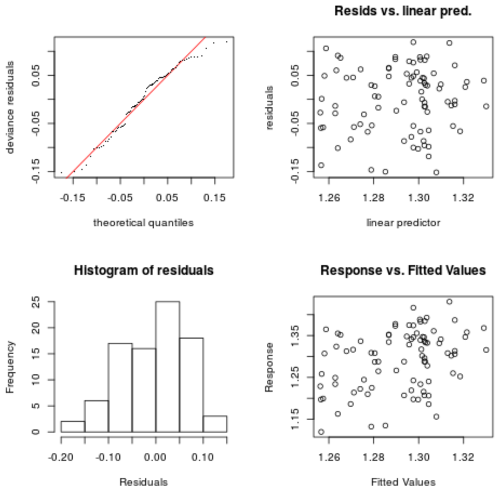

Figure 11: Diagnostics for Model 1 for PMI at the SA2 level.

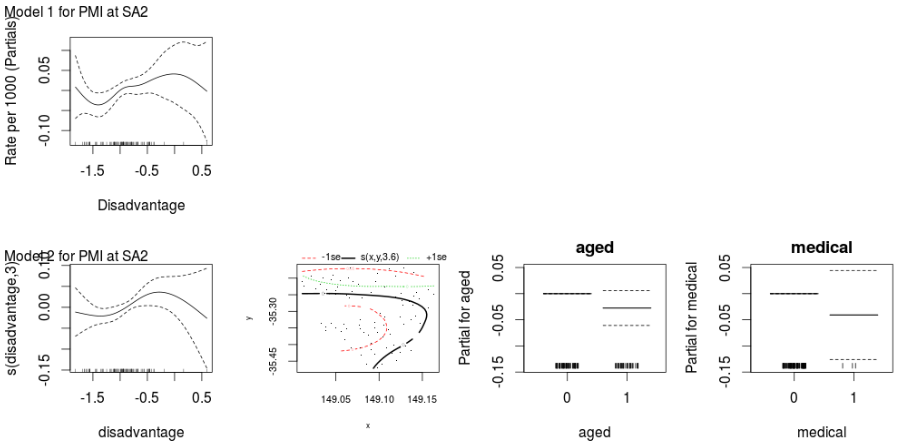

Figure 12: Comparison of predictors in Model 1 and Model 2 for PMI at the SA2 level.

When Model 1 is compared to Model 2 in Figure 12 the exposure-response curves for disadvantage are almost identical. At the scale of SA2 the association with aged village is evident, although this is not statistically significant.

Assessment of residual spatial autocorrelation using semi-variograms

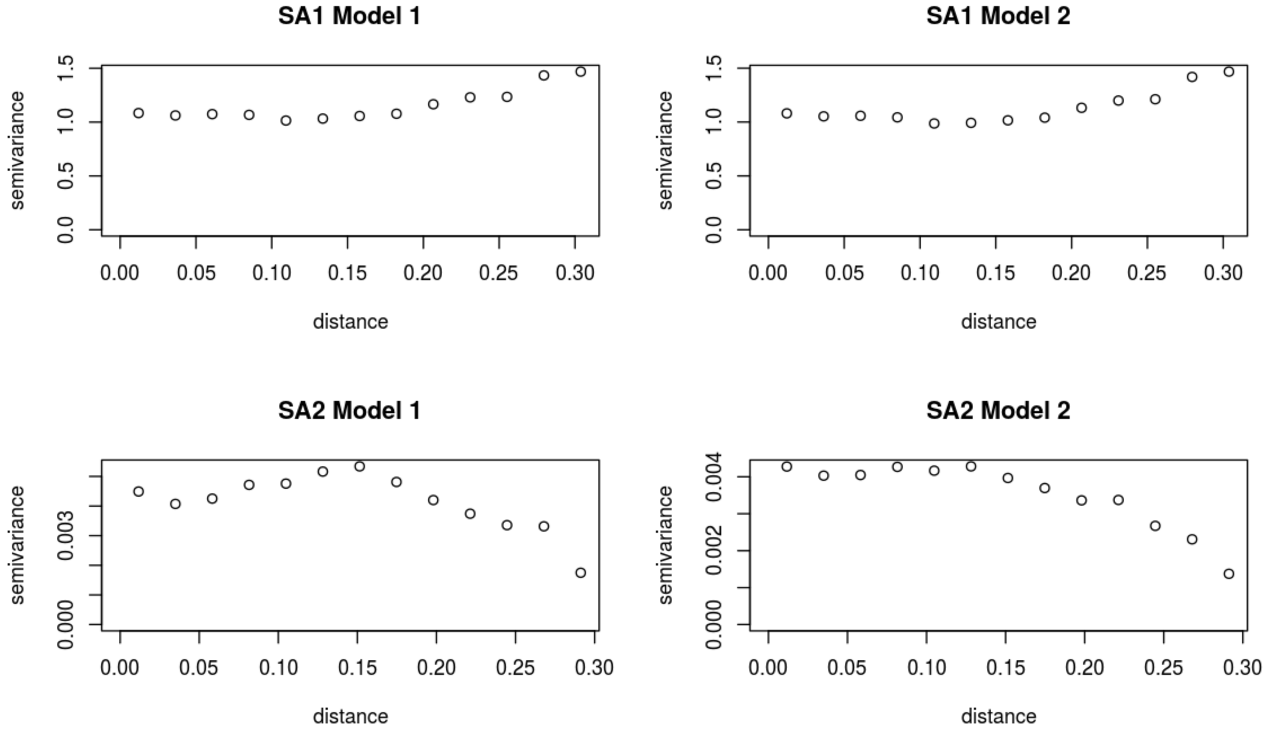

Figure 13: PMI Model 1 and Model 2 variograms of spatial autocorrelation in the residuals.

Figure 13 suggests Model 1 and Model 2 both show no residual spatial autocorrelation. The declining slope at the right-hand side of the SA2 graphs implies some residual autocorrelation at a distance lag of around 0.3 decimal degrees (around 30 kilometers). However, this is likely due to the broad spatial trend of higher rates in the northern-most and southern-most parts of the city (known as a ‘hole-effect’) and would not impact on the statistical quantities being estimated.

SA1 global test of residual spatial autocorrelation

	I	E(I)	var(I)	St. deviate	p-value
PMI, Model 1, Queen, Order 1	-0.0043	-0.0012	0.0004	-0.1447	0.5575
PMI, Model 1, Queen, Order 2	-0.0119	-0.0012	0.0002	-0.7089	0.7608
PMI, Model 1, Queen, Order 3	0.0208	-0.0012	0.0002	1.7403	0.0409
PMI, Model 1, Rook, Order 1	-0.0164	-0.0012	0.0005	-0.6880	0.7543
PMI, Model 1, Rook, Order 2	-0.0052	-0.0012	0.0003	-0.2491	0.5984
PMI, Model 1, Rook, Order 3	0.0074	-0.0012	0.0002	0.6305	0.2642
PMI, Model 2, Queen, Order 1	-0.0293	-0.0012	0.0004	-1.3257	0.9075
PMI, Model 2, Queen, Order 2	-0.0321	-0.0012	0.0002	-2.0500	0.9798
PMI, Model 2, Queen, Order 3	0.0062	-0.0012	0.0002	0.5886	0.2780
PMI, Model 2, Rook, Order 1	-0.0416	-0.0012	0.0005	-1.8215	0.9657
PMI, Model 2, Rook, Order 2	-0.0252	-0.0012	0.0003	-1.4979	0.9329
PMI, Model 2, Rook, Order 3	-0.0064	-0.0012	0.0002	-0.3853	0.6500
PMI, Model 1, Band 0-1	-0.0099	-0.0012	0.0002	-0.5658	0.7142
PMI, Model 1, Band 1-2	0.0237	-0.0012	0.0001	2.5537	0.0053
PMI, Model 1, Band 2-3	-0.0037	-0.0012	0.0001	-0.2940	0.6156
PMI, Model 2, Band 0-1	-0.0314	-0.0012	0.0002	-1.9625	0.9751
PMI, Model 2, Band 1-2	0.0083	-0.0012	0.0001	0.9778	0.1641
PMI, Model 2, Band 2-3	-0.0196	-0.0012	0.0001	-2.1905	0.9858

Table 4: Global Moran's I tests for SA1 with different adjacency definitions, spatial lags (Order) and distance bands (kilometers)

SA1 local indicators of spatial association in residuals

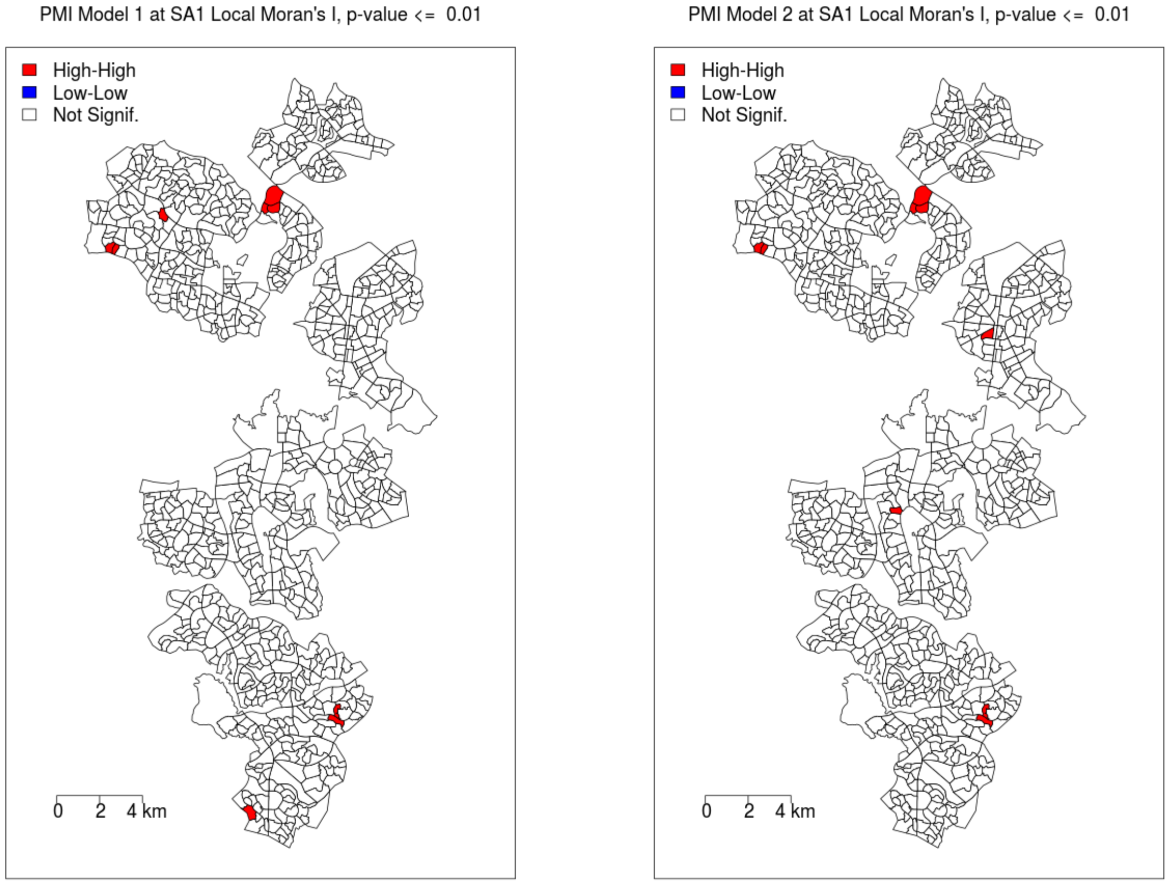

Figure 14: PMI Model 1 and Model 2 at SA2 LISA maps of significantly High-High (red) clustered residuals with p-values less than or equal to 0.01 using first order Queen neighbors. There were no Low-Low (blue) clusters.

SA2 global test of residual spatial autocorrelation

	I	E(I)	var(I)	St. deviate	p-value
PMI, Model 1, Queen, Order 1	-0.0284	-0.0118	0.0047	-0.2411	0.5953
PMI, Model 1, Queen, Order 2	0.0279	-0.0120	0.0033	0.6994	0.2421
PMI, Model 1, Queen, Order 3	-0.0142	-0.0125	0.0036	-0.0278	0.5111
PMI, Model 1, Rook, Order 1	-0.0158	-0.0118	0.0058	-0.0527	0.5210
PMI, Model 1, Rook, Order 2	-0.0236	-0.0118	0.0038	-0.1923	0.5762
PMI, Model 1, Rook, Order 3	0.0384	-0.0125	0.0038	0.8271	0.2041
PMI, Model 2, Queen, Order 1	-0.1325	-0.0118	0.0047	-1.7532	0.9602
PMI, Model 2, Queen, Order 2	-0.0086	-0.0120	0.0033	0.0599	0.4761
PMI, Model 2, Queen, Order 3	-0.0850	-0.0125	0.0036	-1.2147	0.8878
PMI, Model 2, Rook, Order 1	-0.0968	-0.0118	0.0058	-1.1187	0.8684
PMI, Model 2, Rook, Order 2	-0.0941	-0.0118	0.0038	-1.3330	0.9087
PMI, Model 2, Rook, Order 3	-0.0102	-0.0125	0.0038	0.0378	0.4849
PMI, Model 1, Band 0-2	-0.0120	-0.0122	0.0075	0.0026	0.4990
PMI, Model 1, Band 2-4	0.0227	-0.0118	0.0028	0.6526	0.2570
PMI, Model 1, Band 4-6	0.0780	-0.0118	0.0023	1.8842	0.0298
PMI, Model 2, Band 0-2	-0.1064	-0.0122	0.0075	-1.0900	0.8621
PMI, Model 2, Band 2-4	-0.0799	-0.0118	0.0028	-1.2918	0.9018
PMI, Model 2, Band 4-6	0.0141	-0.0118	0.0023	0.5438	0.2933

Table 5: Global Moran's I tests for SA2 with different adjacency definitions, spatial lags (Order) and distance bands (kilometers)

SA2 local indicators of spatial association in residuals

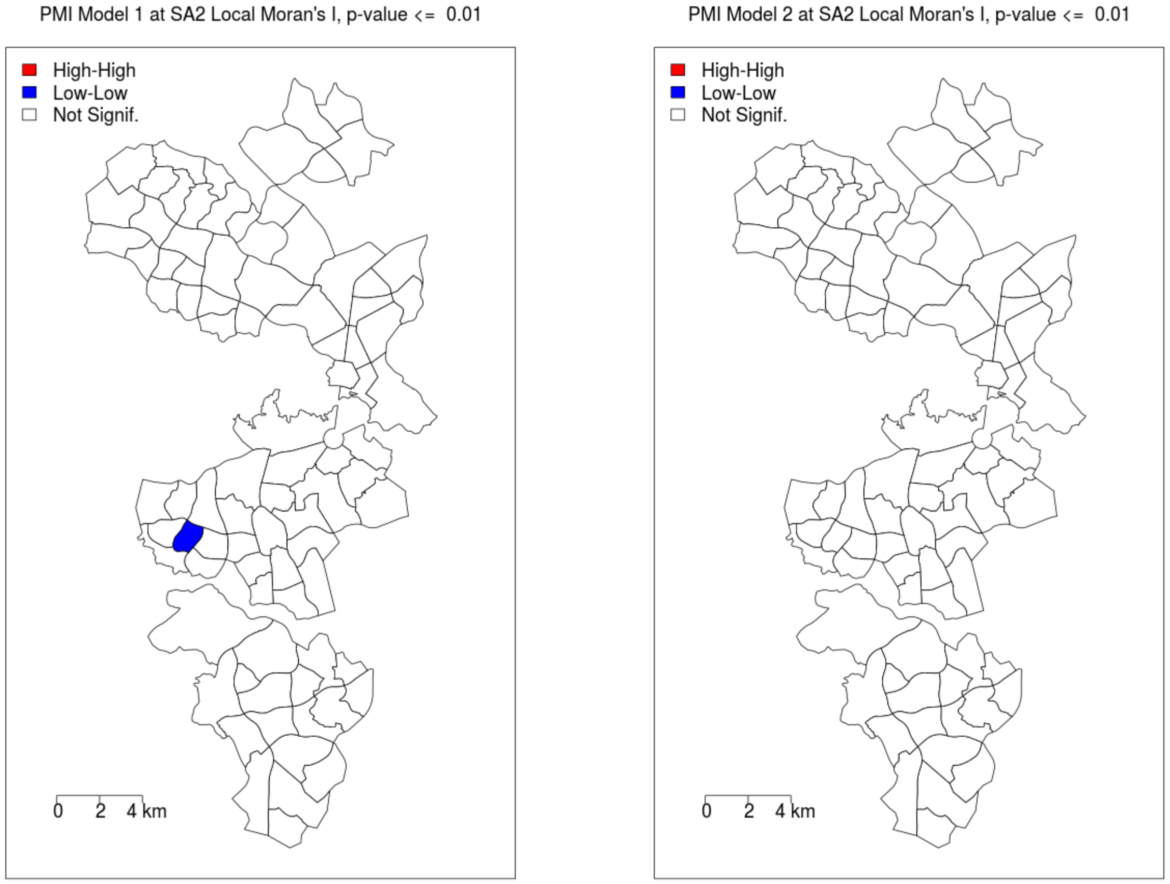

Figure 15: PMI Model 1 and Model 2 at SA2 LISA maps of significantly High-High (red) clustered residuals with p-values less than or equal to 0.01 using first order Queen neighbors. There were no Low-Low (blue) clusters.

PMI SA1 vs SA2

In Figure 16 the fitted disadvantage response curves from Model 1 and Model 2 are shown. These are partial residual curves that show the response function for that term given all other terms being held equal.

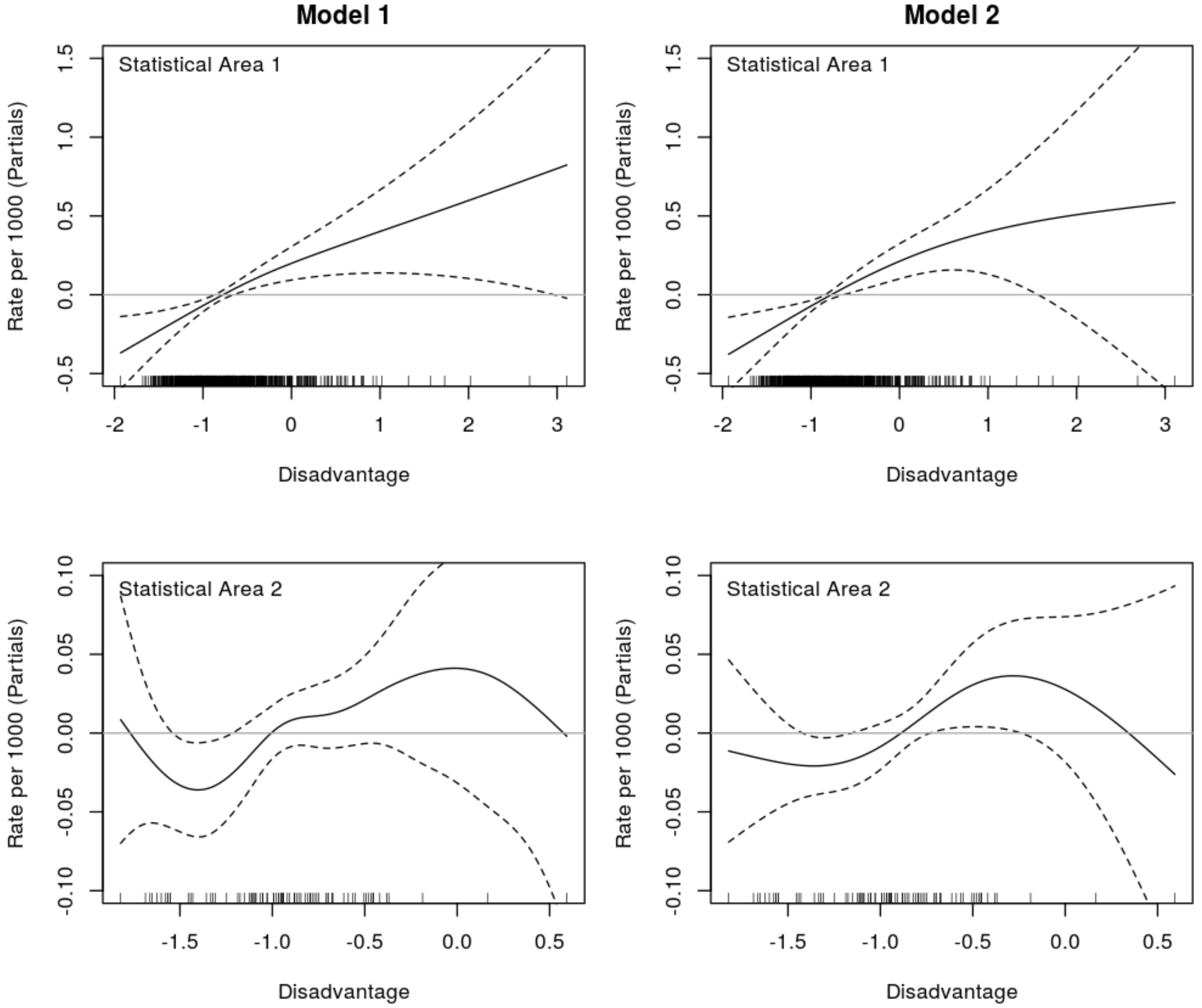

Figure 16: Comparison of Model 1 and Model 2 for PMI at SA1 and SA2 level.

Reproducibility statement

All data preparation tasks were coded so as to ensure reproducibility. Data management was conducted in free and open source software including PostgreSQL/PostGIS, R and visualizations used the free and open source QGIS, except for the final maps which were made with ESRI ArcGIS. All data are available on request, subject to ethical approvals.

All analyses were performed using R statistical language and environment (http://www.r-project.org). Analytical codes are published as open source software at https://bitbucket.org/ivanhanigan/impact-of-scale-manuscript.

The versions of all R packages used to fit the models and write the report are shown below.

```
sessionInfo()
```

```
## R version 3.4.1 (2017-06-30)
## Platform: x86_64-pc-linux-gnu (64-bit)
## Running under: Ubuntu 16.04.3 LTS
##
## Matrix products: default
## BLAS: /usr/lib/libblas/libblas.so.3.6.0
## LAPACK: /usr/lib/lapack/liblapack.so.3.6.0
##
## locale:
##  [1] LC_CTYPE=en_AU.UTF-8       LC_NUMERIC=C
##  [3] LC_TIME=en_AU.UTF-8        LC_COLLATE=en_AU.UTF-8
##  [5] LC_MONETARY=en_AU.UTF-8    LC_MESSAGES=en_AU.UTF-8
##  [7] LC_PAPER=en_AU.UTF-8       LC_NAME=C
##  [9] LC_ADDRESS=C               LC_TELEPHONE=C
## [11] LC_MEASUREMENT=en_AU.UTF-8 LC_IDENTIFICATION=C
##
## attached base packages:
## [1] stats     graphics  grDevices utils     datasets  methods   base
##
## other attached packages:
##  [1] knitr_1.15.1     zoo_1.7-11       car_2.1-1        foreign_0.8-61
##  [5] geoR_1.7-5.2     rgeos_0.3-2      dplyr_0.4.3.9000 rgdal_1.2-8
##  [9] sqldf_0.4-10     RSQLite_1.0.0    DBI_0.7          gsubfn_0.6-6
## [13] proto_0.3-10     mgcv_1.8-19      nlme_3.1-131     spdep_0.6-13
## [17] Matrix_1.2-11    sp_1.2-4
##
## loaded via a namespace (and not attached):
##  [1] gtools_3.4.1           splines_3.4.1
##  [3] lattice_0.20-35        tcltk_3.4.1
##  [5] expm_0.999-2           htmltools_0.3.5
##  [7] yaml_2.1.13            chron_2.3-45
##  [9] nloptr_1.0.4           stringr_1.0.0
## [11] evaluate_0.10          coda_0.16-1
## [13] SparseM_1.6            quantreg_5.11
```

```
## [15] pbkrtest_0.4-6          parallel_3.4.1
## [17] highr_0.4               Rcpp_0.12.6
## [19] xtable_1.7-4            backports_1.0.4
## [21] gdata_2.13.3            deldir_0.1-7
## [23] lme4_1.1-12             digest_0.6.9
## [25] stringi_0.4-1           gmodels_2.15.4.1
## [27] splancs_2.01-36         grid_3.4.1
## [29] rprojroot_1.1           tools_3.4.1
## [31] LearnBayes_2.15         magrittr_1.5
## [33] MASS_7.3-47             RandomFieldsUtils_0.0.10
## [35] RandomFields_3.1.1      assertthat_0.1
## [37] minqa_1.2.4             rmarkdown_1.2
## [39] R6_2.0.1                boot_1.3-20
## [41] nnet_7.3-12             compiler_3.4.1
```

References

Australian Bureau of Statistics. (2013). *Socio-Economic Indexes for Areas (SEIFA) 2011 - Technical Paper. Cat. No. 2033.0.55.001.* Canberra, Australia.

Bivand, R.S., Pebesma, E. & Gomez-Rubio, V. (2008). *Applied Spatial Data Analysis with R.* Springer New York.

Meade, M. & Earickson, R. (2000). *Medical geography.* Guilford Press, New York.

Wood, S. (2008). Fast stable direct fitting and smoothness selection for generalized additive models. *Journal of the Royal Statistical Society: Series B (Statistical Methodology)*, 70(3),: 495–518.

Wood, S. (2006). *Generalized Additive Models: An Introduction with R.* Chapman; Hall/CRC, Boca Raton, USA.
